# *PCD*’s First Annual Student Research Contest: Lui and Wallace Examine Hospitalization Rates for At-Risk Populations

**Published:** 2011-08-15

**Authors:** Samuel F. Posner

**Affiliations:** National Center for Chronic Disease Prevention and Health Promotion, Centers for Disease Control and Prevention

I am pleased to announce that A Common Denominator: Calculating Hospitalization Rates for Ambulatory Care–Sensitive Conditions in California by Camillia K. Lui and Steven P. Wallace is the winner of the first annual *Preventing Chronic Disease (PCD)* Student Research Contest. Ms Lui is a fourth-year doctoral student at the University of California, Los Angeles, in the Department of Community Health Sciences. Her advisor is Dr Steven Wallace.

PCD is dedicated to being the venue for sharing advances in public health research, practice, and policy, and we are committed to the development of young public health professionals as part of this effort. To this end, we have instituted the Student Research Contest as a way to engage students in the publication process and recognize the outstanding work of the next generation of the public health workforce. In July 2010, we announced our first annual call for student papers and reached out to multiple partners to distribute the call and encourage students to submit their work to *PCD*.

Papers were due in January 2011, and we received submissions on a range of topics from institutions throughout the United States. In February and March, a small team of editorial board members (Drs Bowman, Brownson, Lengerich, and Remington), *PCD*’s founding editor (Dr Lynne Wilcox), and I reviewed the submitted manuscripts. In March we selected the paper by Ms Lui and Dr Wallace as the winner.

Lui and Wallace examined the prevalence, hospitalization rates, and geographic variability of hypertension and congestive heart failure, 2 chronic health conditions that are considered to be manageable with effective outpatient treatment (ie, ambulatory care–sensitive conditions), in California. Their analysis makes use of 2 large datasets, the California Health Interview Survey (www.chis.ucla.edu/) and hospital patient discharge files of the California Office of Statewide Health Planning and Development (www.oshpd.ca.gov/). This analysis is important to health care resource planning because it uses the population at risk rather than the total population in calculating hospitalization rates.

With these 2 common conditions, 2 different scenarios emerged. In the case of hypertension, approximately 74% of the geographic areas did not change in ranked quintile when comparing age and age/disease prevalence rates, which suggests that resources are meeting the needs of the at-risk population. However, in the case of congestive heart failure, 31 of the 55 geographic areas in California changed quintile rank — approximately 72% by 2 or more ranks. In this case, the geographic distribution of the population at risk does not mirror that of the general population. This finding has implications for planning and targeting public health programs and health care services in areas where the population at risk resides.

As with any analysis that uses administrative and self-reported data, this study has limitations, and the results do not identify the one area that should be changed to inform policy and programs to reduce health care spending and rates of illness and death. This analysis is thoughtful and identifies several issues that need further investigation. What are the reasons for increased rates of age/risk-adjusted hospitalizations in some geographic areas? Are these observed differences a function of demographic, economic, geographic, or access factors? What are the interventions needed to ensure that people receive adequate and appropriate care? This analysis demonstrates the need to consider disease prevalence when examining hospitalization rates and that, depending on the condition, resources may need to be redistributed.

Congratulations to Ms Lui on winning the first annual PCD Student Research Contest. Several outstanding papers were submitted, and the decision was not an easy one. We thank all of the students who submitted papers for this contest and recognize the hard work that goes into publishing scientific work. Please listen to the short podcast with Ms Lui to hear her discuss her article. Manuscripts for the second annual PCD Student Research Contest are now being accepted, and the winning manuscript will be published in 2012.

**Figure:**
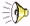
Listen to an interview with Camillia Lui, winner of the inaugural Preventing Chronic Disease Student Paper Competition. (MP3 1.07Mb)

FRAN KRITZ I’m Fran Kritz for *Preventing Chronic Disease*. We’re speaking by phone with Camillia Lui, a graduate student at the UCLA School of Public Health. Camillia Lui is the winner of the inaugural *Preventing Chronic Disease* Student Paper Competition. Camillia, tell us about your paper. What was the title and what were your findings?
CAMILLIA LUI This is quite an honor to get this award, so I’m very appreciative to the journal. The title of the article is “A Common Denominator: Calculating Hospitalization Rates for Ambulatory Care–Sensitive Conditions in California.” I had the opportunity to work with Steven Wallace at the UCLA Center for Health Policy Research on this — on this paper. It was actually a grant received from the California HealthCare Foundation to examine the burden of chronic diseases in California. So, 1 of the ways to examine the burden of chronic disease is to use preventable hospitalization rates. If an area has good access to quality primary care services, the idea is that then there are lower hospitalization rates. So, we’re using that as our key variable for this paper.
FRAN KRITZ And what — tell us the methodology that you used to study the target areas.
CAMILLIA LUI So, we decided to use 2 ways of calculating the hospitalization rates, the traditional way of crude and age-adjusting versus a disease-prevalence method. And the traditional way [is you] usually use the total population in the county as the denominator to calculate the rate. But not everyone is at risk for a hospitalization, especially those who have been diagnosed with a chronic condition. So, we’re fortunate enough to merge in data from the California Health Interview Survey to this data to capture the disease prevalence. So, to capture those who have been diagnosed with a chronic condition, so we used that as the denominator for calculating the rates. And, for this paper, we actually compared the rates using 2 chronic conditions that are manageable with outpatient care, and that was hypertension and congestive heart failure.
FRAN KRITZ And what is the significance of your findings? How do they relate to community care?
CAMILLIA LUI Well, it’s kind of interesting because when we looked at the hypertension rates, it doesn’t matter which method we used. There were very few differences when we compared it by county rankings, but when we were looking at the congestive heart failure, there were actually larger differences with the county rankings such that there are certain counties that just showed a higher burden, using the prevalence-adjusted rate, that wouldn’t have shown that when we looked at the traditional age-adjusted rate. So, it doesn’t mean that 1 way is better than the other, but it does highlight that there might be some underlying differences that traditional methods for calculating hospitalization rates — that they won’t capture.
FRAN KRITZ What was it like working with a mentor?
CAMILLIA LUI It was great. Dr Wallace is definitely for the students, and he gave me the guidance that I needed, but also gave me the sort of freedom to pursue it. We originally did this project for the California HealthCare Foundation, but the next step was to publish it, and through this opportunity, the student contest, we were able to at least submit a manuscript, for it.
FRAN KRITZ And, what’s next for you? What will you be studying?
CAMILLIA LUI So, 1 of the next steps is we are actually going to take these rates and see if they have different factors that are associated with it — associated with, like, access to care factors. The other thing that was really interesting about this project is that the Healthcare Foundation — California HealthCare Foundation, they took the data and were able to make it into this very user friendly, interactive website. So, folks from the county level to public health researchers, to policy makers, they can actually go to this website and click on this — the California county and see what the — what the data look like for chronic disease for their area. So, that was 1 thing that I really enjoyed with this, being able to take data, information about chronic disease and make it readily available and user friendly.
FRAN KRITZ What do you see you doing down the road with your career in public health in addition to the really fine research you’re doing right now?
CAMILLIA LUI I’m definitely a research nerd. One of the things that drove me back to school is to gain the quantitative skills, but to be able to take that back to the community and especially work with community-based organizations with that. There’s just so much data out there, whether it’s community organizations collecting it for themselves or using data such as California Health Interview Survey data to better inform their own work, documents, problems, and strengths of their communities, and inform them for better programs and implementation and service delivery.
FRAN KRITZ Sounds exciting. Camillia Lui, winner of the inaugural *Preventing Chronic Disease* Student Paper Competition, congratulations. I have no doubt we’re hearing — we’ll be hearing lots, both about your research and other activities in the field of public health in the future. Thank you so much.
CAMILLIA LUI Thank you, Fran.
FRAN KRITZ I’m Fran Kritz for *Preventing Chronic Disease*.

